# Enhanced Risk Stratification of Atrial Fibrillation Detected After Ischemic Stroke and TIA: The Role of Apnoea–Hypopnoea Index and Hypoxic Burden—A Study From the Bern Sleep‐Stroke Registry

**DOI:** 10.1111/jsr.70338

**Published:** 2026-04-26

**Authors:** Xiaoli Yang, Julian Lippert, Irina Filchenko, Christian M. Horvath, Sebastien Baillieul, Corrado Bernasconi, Stefan A. Bauer‐Gambelli, Anne‐Kathrin Brill, David J. Seiffge, Elias Auer, Tobias Reichlin, Urs Fischer, Marcel Arnold, Markus H. Schmidt, Claudio L. A. Bassetti

**Affiliations:** ^1^ Department of Neurology, Inselspital Bern University Hospital, University of Bern Bern Switzerland; ^2^ Interdisciplinary Sleep‐Wake‐Epilepsy‐Center Inselspital, Bern University Hospital, University of Bern Bern Switzerland; ^3^ Department of Pulmonary Medicine, Allergology and Clinical Immunology, Inselspital Bern University Hospital, University of Bern Bern Switzerland; ^4^ HP2 Laboratory, INSERM U1300, Grenoble Alpes University Hospital Grenoble Alpes University Grenoble France; ^5^ Department of Cardiology, Inselspital Bern University Hospital, University of Bern Bern Switzerland; ^6^ University of Bern and Department of Neurology, Inselspital, Bern University Hospital Bern Switzerland

**Keywords:** acute ischemic stroke, cardiac arrhythmias, sleep apnoea syndromes

## Abstract

Sleep‐disordered breathing is an independent risk factor for stroke and atrial fibrillation. Accurate assessment of atrial fibrillation in stroke patients with sleep‐disordered breathing is crucial for secondary prevention. This study aimed to determine whether combining apnoea–hypopnoea index and hypoxic burden improves risk stratification for atrial fibrillation detected after ischemic stroke. About 911 patients with ischemic stroke underwent respiratory polygraphy within 3 days after an event. Hypoxic burden was defined as the cumulative area under the oxygen desaturation curve of respiratory events. Atrial fibrillation was monitored with up to three 7‐day electrocardiogram recordings within 6 months. Patients were stratified by apnoea–hypopnoea index (≥ 15 vs. < 15 events/h) and by hypoxic burden (above vs. below the median, 35% min h^−1^). Logistic regression adjusted for age, sex, and cardiovascular risk factors assessed associations between apnoea–hypopnoea index/hypoxic burden categories and atrial fibrillation. Among 911 patients (mean age 66 ± 14 years, 62% male), atrial fibrillation was detected in 145 (16%) patients. Patients with both apnoea–hypopnoea index ≥ 15 and high hypoxic burden had nearly double the odds of atrial fibrillation compared to those with low apnoea–hypopnoea index and low hypoxic burden (adjusted odds ratio 1.97, 95% CI 1.25–3.12). In contrast, neither elevated apnoea–hypopnoea index nor high hypoxic burden alone demonstrated a statistically significant association. Elevated apnoea–hypopnoea index combined with high hypoxic burden identifies stroke patients at higher risk of atrial fibrillation. Integrating these metrics into post‐stroke evaluation may improve personalized management and secondary prevention of recurrent cardiovascular or cerebrovascular events.

**Abbreviations**
AFDAS: atrial fibrillation detected after stroke
CE: cardioembolic stroke
CPAP: continuous positive airway pressure
HB: hypoxic burden
LAA: large artery atherosclerosis
NIHSS: National Institute of Health Stroke Scale
SDB: sleep‐disordered breathing
SSR: Swiss Stroke Registry
SVO: small vessel occlusion
TIA: transient ischemic attack

## 1 Introduction

Sleep‐disordered breathing (SDB) is highly prevalent, affecting approximately 70% of acute stroke patients (Baillieul et al. 2022; Hasan et al. 2021). It is also recognized as an independent risk factor for cerebro‐cardiovascular diseases (CCVE) (Yaggi et al. 2005). Furthermore, the presence and severity of SDB have been independently linked to the development of atrial fibrillation (AF) (Cadby et al. 2015).

AF, the most common sustained cardiac arrhythmia, increases stroke risk by five‐fold (Seet et al. 2011) and it is responsible for approximately 25% of ischemic strokes, predominantly due to cardio‐embolic causes (Murtagh and Smalling 2006). Importantly, SDB is also an independent predictor of stroke in patients with AF (Yaranov et al. 2015).

In the context of acute ischemic stroke, comorbid moderate‐to‐severe SDB (apnoea–hypopnoea index [AHI] ≥ 15/h) and AF is frequent and significantly worsens outcomes, leading to a 2.5‐fold increase in long‐term mortality or recurrent CCVE (Yang et al. 2024).

The frequent coexistence of SDB and AF is attributed to shared risk factors and converging pathophysiological mechanisms, including intermittent hypoxia, which triggers oxidative stress and inflammation; intrathoracic pressure shifts that lead to cardiac strain; autonomic dysregulation promoting arrhythmogenesis; and structural atrial remodelling that facilitates the persistence of AF (Iwasaki et al. 2014; Linz et al. 2021, 2022, 2018; Monahan et al. 2009; Goudis and Ketikoglou 2017).

However, AF in ischemic stroke is often undetected due to its short duration, episodic occurrences, and frequently asymptomatic nature, making bedside diagnosis challenging and leading to suboptimal secondary prevention (Seet et al. 2011).

SDB is commonly quantified using the AHI, which measures the frequency of apnoeic and hypopnoeic events per hour of sleep. Although AHI is widely used to diagnose the severity of SDB, it has significant limitations in characterizing the pathophysiological mechanisms linking SDB and AF, particularly in stroke patients. AHI does not adequately reflect the duration, depth, or cumulative impact of apnoeic episodes and oxygen desaturation, all of which are critical factors influencing cardiovascular outcomes (Seiler et al. 2019).

To overcome AHI's limitations, HB was introduced, capturing the frequency, duration, and severity of hypoxic episodes for a more comprehensive cerebro‐cardiovascular risk assessment. HB has shown promise in identifying high cardiovascular risk individuals across various cohorts (Azarbarzin et al. 2020, 2019; Pinilla et al. 2023; Trzepizur et al. 2022). Nevertheless, relying solely on HB may still fall short of optimal risk stratification.

A combined approach that integrates both AHI and HB could more accurately reflect the multifaceted cardiovascular impact of SDB. This integrated method has the potential to improve risk stratification and guide secondary prevention strategies, particularly for stroke patients, by addressing the complex interplay between SDB and AF.

We aimed to test whether combining AHI with HB improves risk stratification for atrial fibrillation detected after stroke (AFDAS) compared with either metric alone. We hypothesized that the coexistence of elevated AHI and HB would identify the SDB phenotype with the highest odds of AFDAS, whereas each metric alone would demonstrate attenuated associations.

## 2 Methods

### 2.1 Study Design and Participants

The Bern Sleep‐Stroke Registry is a retrospective, observational, single‐center cohort study that is based on clinical data prospectively collected from the Swiss Stroke Registry, a mandatory, prospectively maintained national database. Since 2014, the Swiss Stroke Registry has included all consecutive patients hospitalized in certified stroke units and comprehensive stroke centres across Switzerland, meeting national criteria aligned with the European Stroke Organization standards (Waje‐Andreassen et al. 2018). The registry is designed to support quality control and research in the management of acute stroke (Manno et al. 2019; Bonati et al. 2016). For the present analysis, only patients with ischemic stroke or TIA who were admitted to Inselspital, Bern University Hospital from January 1, 2017, to August 31, 2021 were included. The following data were collected: patient demographics (age, sex, and body mass index), medical history (known ischemic stroke, hypertension, dyslipidaemia, diabetes, AF, coronary heart disease, chronic obstructive pulmonary disease, and heart failure), clinical data (National Institutes of Health Stroke Scale score [NIHSS], blood glucose levels, blood pressure on admission), and diagnostic test results for stroke (imaging modality) and SDB (respiratory polygraphy).

Exclusion criteria for this study included: (1) lack of respiratory polygraphy during acute hospitalization or missing/insufficiently reliable HB data; (2) supplemental oxygen during the recording; and (3) pre‐existing AF or incomplete diagnostic evaluation for AFDAS. Patients without RP were defined as those in whom RP was not performed. Recordings with excessive artefacts or insufficient analysable SpO_2_ or respiratory event signals were classified as unavailable, missing, or insufficiently reliable HB data.

The study was approved by the Ethics Committee of the University of Bern (Protocol number: 2021‐00883). Individual informed consent was waived according to the Swiss law.

In compliance with Swiss law, patients who declined the use of their biological data for research purposes after being informed about its collection were excluded from the analysis. This study adheres to the principles outlined in the Declaration of Helsinki.

### 2.2 Respiratory Polygraphy

Within 3 days after admission and as a part of standard clinical routine at the Stroke Center, Inselspital, patients underwent screening for SDB using respiratory polygraphy (Nox Medical Inc., Reykjavik, Iceland) (Gottlieb and Punjabi 2020). An apnoea was defined as a ≥ 90% reduction in nasal pressure signal, while a hypopnoea was defined as a ≥ 30% reduction in airflow combined with ≥ 3% arterial oxygen desaturation, each lasting at least 10 s. Central sleep apnoea was defined as central apnoea index (CAI) ≥ 5/h, with a CAI/AHI ratio > 0.5 (Schütz et al. 2021). Results of respiratory polygraphies were assessed by an experienced scorer following the guidelines of the American Academy of Sleep Medicine (Berry et al. 2012).

### 2.3 Hypoxic Burden Computation

Hypoxic burden (HB) was computed from the pulse oximetry (SpO_2_) signal and scored respiratory events using a MATLAB‐based implementation of the sleep apnoea–specific HB approach described by Azarbarzin et al. (Azarbarzin et al. 2019). Respiratory events (apnoeas and hypopnoeas; duration ≥ 10 s) were extracted from manually scored respiratory polygraphy recordings. Prior to HB computation, the SpO_2_ signal underwent automated quality control and artefact handling (including removal of physiologically implausible values, flat‐line segments, and abrupt step changes; and exclusion of recordings with insufficient artefact‐free signal). To avoid double counting of a single desaturation across closely spaced events, events separated by < 20 s were merged prior to AUC attribution.

For each respiratory event, the event‐specific baseline SpO_2_ was defined as the maximum SpO_2_ within the 100 s preceding the end of the event. The desaturation area attributable to that event was then calculated as the time integral of (baseline SpO_2_ − observed SpO_2_) over a window spanning 2 s before event onset to 60 s after event termination, with the integration truncated at the first return to baseline within the window (or at the window limit if baseline was not re‐attained). Event‐level desaturation areas (SpO_2_‐percent × minutes) were summed across all events and normalized by the total analysed recording time (hours), yielding HB in % min h^−1^.

The MATLAB implementation was internally verified within our study by applying the same prespecified pipeline to an independent subset of recordings from the registered “Sleep Deficiency and Stroke Outcome” cohort (NCT02559739). The purpose of this validation was not to directly compare predictive performance across cohorts with different endpoints, but to verify correct implementation and robustness of the signal‐processing pipeline by demonstrating consistent behaviour when applied unchanged to independent recordings.

Similarly, patients were stratified into AHI+ or AHI− groups based on their baseline AHI, with a threshold set at 15/h. Those with an AHI of ≥ 30/h were categorized as having severe SDB (Berry et al. 2012). Patients were stratified into HB+ or HB− groups based on their baseline HB values, utilizing the median HB value (35% min h^−1^) as the threshold (Azarbarzin et al. 2020; Pinilla et al. 2023).

For the analyses, four groups were defined based on distinct combinations of AHI and HB: AHI+/HB+, AHI+/HB−, AHI−/HB+, and AHI−/HB−. These groups were then compared.

### 2.4 Seven‐Day Holter ECG Monitoring

Study participants underwent up to three 7‐day Holter ECG recordings at 2‐month intervals. The initial recording was conducted at the time of hospital discharge, while subsequent recordings were performed on an outpatient basis. If AF was detected during any recording, further scheduled recordings were discontinued. The Lifecard CF Holter system (Spacelabs Healthcare, Issaquah, WA) was employed, which provided continuous two‐channel ECG recording for 7 days. All recordings were systematically analysed for the presence of AF and other incidental arrhythmias using the Pathfinder SL software (Spacelabs Healthcare), supplemented by manual confirmatory analysis to ensure accuracy.

AF was defined in accordance with current clinical guideline (Calkins et al. 2007) as episodes of irregular heart rhythm lasting ≥ 30 s in the absence of identifiable reversible causes. This definition aligns with established diagnostic criteria in the literature, ensuring consistency and comparability across studies.

During the 7‐day monitoring period, patients were instructed to keep detailed diaries. These records provided supplementary data, offering valuable contextual information to enrich the interpretation of monitoring results.

### 2.5 Statistics

Continuous variables are presented as mean ± SD, while categorical variables are expressed as counts with percentages. Comparisons between continuous variables and categorical variables were performed using the Mann–Whitney U, Pearson *χ* ^2^, or Fisher exact tests, as appropriate.

A series of unadjusted and adjusted logistic regression models were employed to evaluate the impact of various combinations of AHI and HB (AHI+/HB+, AHI+/HB−, AHI−/HB+) relative to the reference group AHI−/HB− on the incidence of AFDAS.

Covariates were selected based on well‐established clinical associations with AF (Andrade et al. 2014). The analysis included partially adjusted models, which accounted for age and sex, as well as fully adjusted models that incorporate a comprehensive range of covariates: age, sex, body mass index, smoking status, hypertension, diabetes, dyslipidaemia, coronary heart disease, chronic obstructive pulmonary disease, and heart failure. Odds ratios from both partially and fully adjusted models were compared across the four AHI/HB groups to evaluate the relationship between these SDB phenotypes and AFDAS.

Sample size was determined by the number of consecutive eligible patients with complete respiratory polygraphy and rhythm‐monitoring data. For the primary comparison (AHI+/HB+ vs. AHI−/HB−; *n* = 336 vs. *n* = 404; reference AFDAS incidence 11.4%), a post hoc power estimation suggested adequate power (~96.7%) to detect the observed difference at a two‐sided *α* = 0.05. In addition, with 145 total events and 10 confounders in the multivariable model, the study maintained an events‐per‐variable ratio of approximately 11.15, ensuring the stability of the logistic regression estimates.

Sensitivity analyses were conducted to assess the robustness of associations between SDB severity, nocturnal hypoxemia metrics, and AFDAS. Multivariable logistic regression models were refitted using alternative parameterizations of AHI, which were examined as a continuous variable (untransformed and log_2_‐transformed) and as categorical variables using conventional clinical cutoffs (≥ 5/h, ≥ 15/h, and ≥ 30/h).

Additional sensitivity analyses compared HB with established SpO_2_‐derived metrics. In fully adjusted logistic regression models, nocturnal hypoxemia was alternatively entered as oxygen desaturation index (ODI), HB (log_2_‐transformed), mean nocturnal SpO_2_, lowest SpO_2_, and T90 (percentage of recording time with SpO_2_ < 90%).

To explore whether the association of HB differs across intermittent hypoxia severity, we conducted exploratory ODI‐stratified analyses (ODI ≥ 5 vs. ≥ 15 events h^−1^) by refitting the fully adjusted model within each stratum to assess whether HB provided incremental information beyond ODI.

Statistical significance was set at *p* < 0.05. All analyses were performed in R version 4.0.2 (R Foundation for Statistical Computing; https://www.r‐project.org/) and all signal analyses were performed in MATLAB (MathWorks). This study adheres to the Strengthening the Reporting of OBservational Studies in Epidemiology (STROBE) guideline (Von Elm et al. 2007).

## 3 Results

**FIGURE 1:**
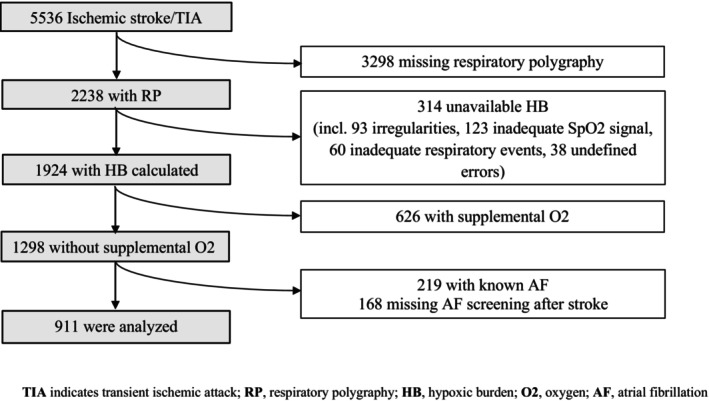
Flow chart of patient's selection. AF, atrial fibrillation; HB, hypoxic burden; O_2_, oxygen; RP, respiratory polygraphy; TIA, transient ischemic attack.

Out of 5536 patients treated for acute stroke or TIA during the study period and enrolled in the SSR, 3298 individuals were excluded due to the lack of respiratory polygraphy. About 314 were excluded because of missing HB calculation, 626 were excluded due to supplemental oxygen during RP screening, 219 known‐AF were excluded, and 168 were excluded due to missing AF monitoring after stroke. Finally, 911 patients were included in the analysis (Figure 1).

Of the 911 patients included in the study, 145 (16%) were diagnosed with AFDAS. Specifically, of the 145 patients diagnosed with AFDAS, 121 (83%) underwent a 7‐day Holter ECG: 57 (47%) patients received three screenings, 30 (25%) underwent two screenings, and 34 (28%) had a single screening. The remaining 24 cases were diagnosed using alternative methods: 15 were identified using 24‐ or 48‐h ECG monitoring during acute care in the Stroke Unit, eight were diagnosed using an implantable loop recorder, and one was detected through a pacemaker readout.

Table 1 presents descriptive statistics of the four combinations of AHI and HB categories (AHI+/HB+, AHI+/HB−, AHI−/HB+, AHI−/HB−). The AHI+/HB+ group was predominantly male and characterized by a higher BMI, AHI, ODI, and CAI. This group also had a greater prevalence of severe SDB (AHI ≥ 30/h) and central sleep apnoea.

**TABLE 1:** Baseline characteristics.

	AHI−/HB− (*N* = 404)	AHI+/HB− (*n* = 54)	AHI−/HB+ (*n* = 117)	AHI+/HB+ (*n* = 336)	Total (*n* = 911)	*p*
Age, years	62.5 (14.9)	67.5 (12.3)	68.0 (11.2)	69.5 (11.9)	66.1 (13.6)	< 0.001
Male sex, *n* (%)	214 (53.0%)	40 (74.1%)	62 (53.0%)	243 (72.3%)	559 (61.4%)	< 0.001
BMI (kg m^−2^)	25.3 (4.1)	26.6 (3.3)	26.3 (4.3)	27.1 (4.7)	26.2 (4.4)	< 0.001
Smoking status *n* (%)	128 (31.7%)	16 (29.6%)	35 (29.9%)	104 (31.1%)	283 (31.1%)	0.978
Sleep features						
AHI (events h^−1^)	5.1 (3.4)	26.2 (11.1)	9.9 (3.8)	34.4 (17.1)	17.8 (17.5)	< 0.001
ODI (events h^−1^)	6.3 (4.2)	16.2 (11.4)	15.2 (12.0)	30.7 (16.2)	17.0 (15.8)	< 0.001
AHI ≥ 30/h, *n* (%)	0 (0.0%)	16 (29.6%)	0 (0.0%)	166 (49.4%)	182 (20.0%)	< 0.001
CAI (events h^−1^)	0.4 (0.8)	3.0 (4.5)	1.6 (4.1)	6.0 (10.2)	2.8 (7.0)	< 0.001
OAI (events h^−1^)	0.9 (1.3)	5.3 (5.7)	3.1 (4.9)	9.3 (9.7)	4.5 (7.4)	< 0.001
OAI ≥ 5/h, *n* (%)	7 (1.7%)	21 (38.9%)	20 (17.1%)	197 (58.6%)	245 (26.9%)	< 0.001
CAI ≥ 5/h, *n* (%)	2 (0.5%)	11 (20.4%)	7 (6.0%)	99 (29.5%)	119 (13.1%)	< 0.001
CSA (events h^−1^)	47 (11.9%)	16 (30.8%)	18 (15.7%)	78 (23.4%)	159 (17.8%)	< 0.001
Event characteristics, *n* (%)						
Initial NIHSS	3.1 (4.5)	3.5 (4.9)	3.8 (5.5)	3.7 (4.5)	3.4 (4.7)	0.175
TOAST						< 0.001
CE	34 (8.4%)	3 (5.6%)	13 (11.1%)	125 (37.2%)	175 (19.2%)	
LAA	72 (17.8%)	9 (16.7%)	20 (17.1%)	65 (19.3%)	166 (18.2%)	
SVO	34 (8.4%)	2 (3.7%)	15 (12.8%)	28 (8.3%)	79 (8.7%)	
Other	85 (21.0%)	14 (25.9%)	16 (13.7%)	38 (11.3%)	153 (16.8%)	
Unknown	179 (44.3%)	26 (48.1%)	53 (45.3%)	80 (23.8%)	338 (37.1%)	
Supratentorial	317 (78.5%)	41 (75.9%)	87 (74.4%)	285 (84.8%)	730 (80.1%)	0.039
Infratentorial	102 (25.2%)	13 (24.1%)	33 (28.2%)	56 (16.7%)	204 (22.4%)	0.014
Event type, *n* (%)						
Ischemic stroke	349 (86.4%)	51 (94.4%)	104 (88.9%)	300 (89.3%)	804 (88.3%)	0.29
TIA	5 (13.6%)	3 (5.6%)	13 (11.1%)	36 (10.7%)	107 (11.7%)	
Cerebro‐cardiovascular risk factors, *n* (%)						
Hypertension	221 (54.7%)	35 (64.8%)	94 (80.3%)	266 (79.2%)	616 (67.6%)	< 0.001
Diabetes	52 (12.9%)	10 (18.5%)	26 (22.2%)	84 (25.0%)	172 (18.9%)	< 0.001
Dyslipidaemia	256 (63.4%)	36 (66.7%)	82 (70.1%)	238 (70.8%)	612 (67.2%)	0.161
CHD	37 (9.2%)	6 (11.1%)	13 (11.1%)	68 (20.2%)	124 (13.6%)	< 0.001
Heart failure	10 (2.5%)	2 (3.7%)	7 (6.0%)	34 (10.1%)	53 (5.8%)	< 0.001
COPD	6 (1.5%)	0 (0.0%)	3 (2.6%)	5 (1.5%)	14 (1.5%)	0.643
AFDAS	46 (11.4%)	8 (14.8%)	18 (15.4%)	73 (21.7%)	145 (15.9%)	0.002

*Note:* Data are presented as mean ± standard deviation (SD) or *n* (%).

Abbreviations: AFDAS, atrial fibrillation detected after stroke; AHI, apnoea–hypopnoea index; BMI, body mass index; CAI, central apnoea index; CE, cardioembolic stroke; CHD, coronary heart disease; COPD, chronic obstructive pulmonary disease; CSA, central sleep apnoea; LAA, large‐artery atherosclerosis; NIHSS, National Institute of Health Stroke Scale; OAI, obstructive apnoea index; ODI, oxygen desaturation index; SVO, small vessel occlusion; TIA, transient ischemic attack; TOAST, Trial of Org 10172 in Acute Stroke Treatment.

In terms of acute ischemic stroke/TIA characteristics, the AHI+/HB+ group showed the highest proportions of cardioembolic stroke, large artery atherosclerosis, and strokes located in supratentorial regions. Regarding cerebro‐cardiovascular risk factors, this group had the highest rates of pre‐existing diabetes and dyslipidaemia, underscoring significant cerebro‐cardiovascular risk factors.

Importantly, the AHI+/HB+ group showed the highest incidence of AFDAS among the four categories.

To investigate the associations between various combinations of exposures represented by AHI and HB on AFDAS, partially (adjusted for age and sex) and fully adjusted logistic regression models adjusted for age, sex, body mass index, smoking status, hypertension, diabetes, dyslipidaemia, coronary heart disease, chronic obstructive pulmonary disease, and heart failure were performed.

**FIGURE 2:**
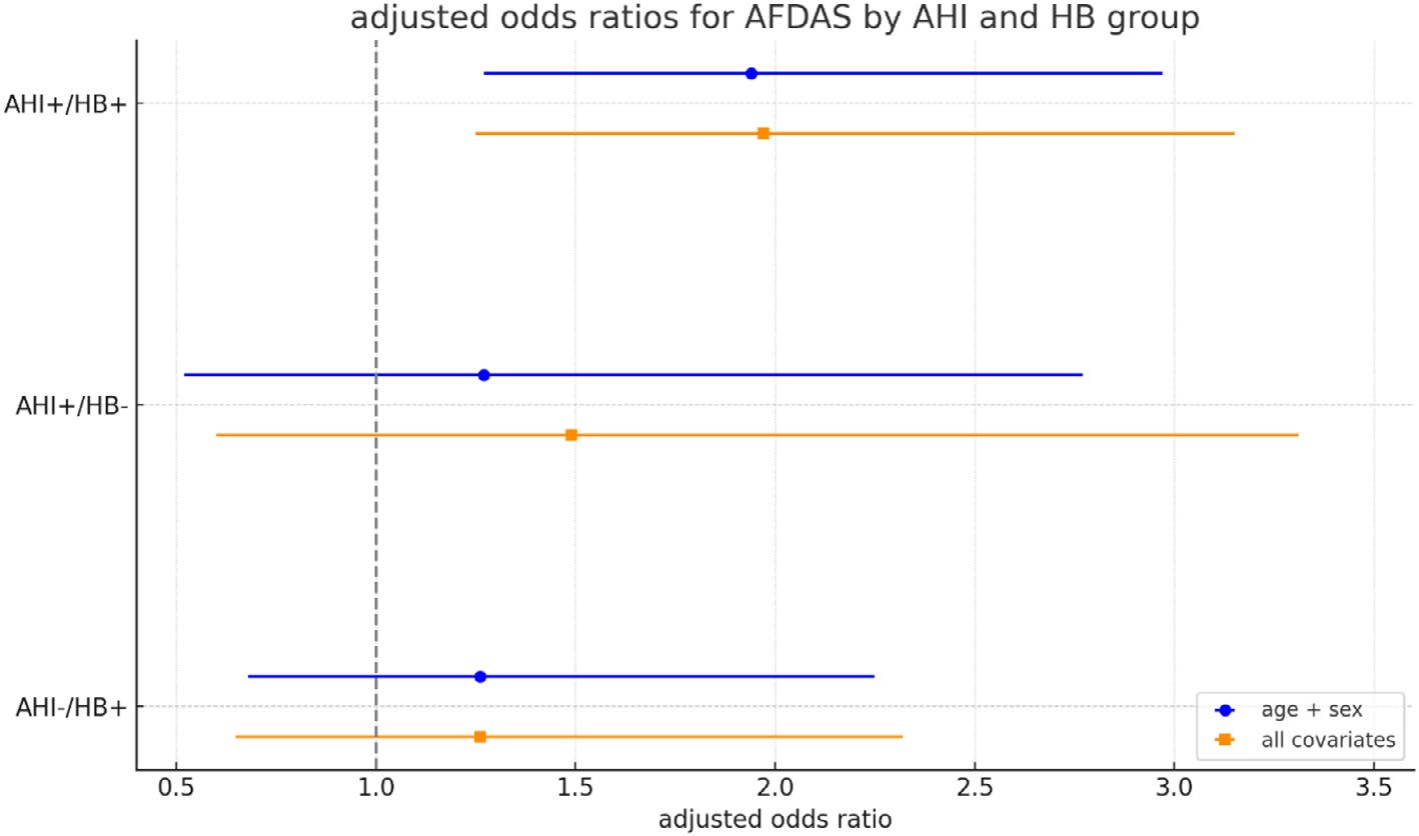
Adjusted odds ratios for AFDAS across distinct combinations of AHI and HB (AHI+/HB+, AHI+/HB−, and AHI−/HB+), in comparison to the reference group AHI−/HB−. AHI was classified into AHI+ (AHI ≥ 15/h) and AHI− (AHI < 15/h). HB was categorized as HB+ (> 35% min h^−1^ [median HB]) and HB− (≤ 35% min h^−1^). AHI indicates apnoea–hypopnoea index, defined as apnoea and hypopnoea events associated with ≥ 3% desaturation. HB, hypoxic burden. All covariates: Age, sex, BMI, smoking, hypertension, diabetes, dyslipidaemia, coronary heart disease, chronic obstructive pulmonary disease, and heart failure.

In the partially adjusted models, compared to the AHI−/HB− (reference), patients with AHI+/HB+ exhibited an odds ratio of 1.94 [95% CI, 1.27–2.97] for AFDAS. Conversely, no significant difference was observed in patients with AHI+/HB− (odds ratio, 1.27 [95% CI, 0.52–2.77]) and in patients with AHI−/HB+ (odds ratio, 1.26 [95% CI, 0.68–2.25]) (Figure 2).

In the fully adjusted model, when compared to the AHI−/HB−, patients with AHI+/HB+ exhibited an odds ratio of 1.97 [95% CI, 1.25–3.15]. Similarly, no significant difference was observed in patients with AHI+/HB− (odds ratio, 1.49 [95% CI: 0.60–3.31]) and in patients with AHI−/HB+ (odds ratio, 1.26 [95% CI, 0.65–2.32]) (Figure 2).

To investigate the distinct predictive capabilities of AHI and HB, logistic regression was also utilized to examine the association between each baseline variable and AFDAS. Unadjusted and fully adjusted logistic regression analyses were conducted to evaluate the individual predictive strength of continuous AHI and HB (untransformed and log_2_‐transformed), along with dichotomized AHI (with cutoffs at 5/h, 15/h, and 30/h) and HB (with a cutoff at the median value 35% min h^−1^). In both unadjusted and adjusted regression models, continuous AHI and HB as well as dichotomized AHI and HB exhibited significant associations with AFDAS (Table 2).

**TABLE 2:** Comparative associations of AHI and HB with AFDAS: Unadjusted and fully adjusted logistic regression models.

Measures	Unadjusted OR	95% CI	Adjusted OR	95% CI
AHI	1.02	1.01–1.03	1.02	1.01–1.03
Log‐AHI	1.28	1.12–1.46	1.27	1.09–1.48
AHI+	1.87	1.31–2.68	1.78	1.19–2.67
AHI ≥ 30/h	2.24	1.50–3.31	2.03	1.43–3.07
HB	1.004	1.001–1.007	1.004	1.001–1.008
Log‐HB	1.25	1.09–1.43	1.22	1.05–1.43
HB+	1.88	1.31–2.72	1.64	1.10–2.47

*Note:* All covariates: age, sex, BMI, smoking, hypertension, diabetes, dyslipidaemia, coronary heart diseases, chronic obstructive pulmonary disease, and heart failure.

Abbreviations: AHI, apnoea–hypopnoea index; AHI+, AHI ≥ 15/h; HB, hypoxic burden; HB+, HB ≥ 35% min h^−1^, (cutoff set at median HB); OR, odds ratio.

In covariate‐adjusted analyses, classical saturation‐based measures (mean SpO_2_, lowest SpO_2_, and T90) were not independently associated with AFDAS (Table S2). In contrast, both ODI and HB showed associations with AFDAS when modelled separately, but predictive discrimination was nearly identical when using either ODI or HB as the primary hypoxemia descriptor (AUC ~0.68), indicating that HB did not outperform ODI in this cohort. Consistent with this overlap, ODI and HB were strongly correlated overall, yet HB showed marked heterogeneity at lower ODI values (Figure S2). In exploratory ODI‐stratified models, HB remained independently associated with AFDAS when restricting the analysis to individuals with ODI ≥ 5 events h^−1^ (odds ratio, 1.24 [95% CI, 1.02–1.50]), whereas in the moderate‐to‐severe range (ODI ≥ 15 events h^−1^), HB did not provide incremental predictive value, even in the unadjusted model (odds ratio, 1.10 [95% CI, 0.86–1.42]).

## 4 Discussion

This study evaluated the utility of a combined AHI and HB model to improve AFDAS risk stratification. Patients with concurrently elevated AHI and HB (AHI+/HB+) demonstrated the highest odds of AFDAS relative to those with neither elevated (AHI−/HB−). This association persisted after adjustment for age, sex, and traditional cardiovascular risk factors, underscoring the robustness of this combined metric. Notably, neither high AHI nor high HB alone was independently associated with AFDAS.

From a pathophysiological perspective, this finding underscores the cumulative cardiac stress imposed by frequent obstructive events combined with profound and sustained oxygen desaturations. Recurrent obstructive apnoeas induce large intrathoracic pressure swings that stretch the atrial walls, contributing to structural remodelling, and each apnoea termination triggers surges in sympathetic activity that can precipitate atrial ectopic beats (Teba and Yaggi 2013). At the same time, chronic intermittent hypoxemia leads to oxidative stress and systemic inflammation, creating an electrophysiological substrate that favours AF onset (Saleeb‐Mousa et al. 2023). The combination of frequent respiratory events and a high HB imposes cumulative cardiac stress. This may cross the threshold required for AF initiation, whereas either factor alone might be insufficient. Consistent with this, a large cohort study recently demonstrated that sleep‐related hypoxemia is a stronger predictor of incident AF than AHI: a 10% decrease in mean nocturnal oxygen saturation was associated with ~30% higher AF risk, whereas a 10‐event/h increase in AHI conferred only ~2% higher risk (Heinzinger et al. 2023). In stroke patients, greater nocturnal oxygen desaturation has likewise been independently linked to higher odds of AF (Teba and Yaggi 2013), reinforcing the role of HB in AFDAS.

Additionally, the AHI+/HB+ group exhibited the highest prevalence of cardioembolic strokes (37.2%), consistent with a higher likelihood of underlying AF in these patients (Arboix and Alió 2010). This underscores the importance of vigilant ECG monitoring and appropriate secondary prevention in this high‐risk subgroup. Our study represents the largest acute stroke cohort to date assessing the combined prognostic value of AHI and HB for AFDAS.

Emerging research has examined the individual roles of AHI and HB in predicting CCVE. In many studies, HB correlates more strongly with CCVE than AHI (Azarbarzin et al. 2020, 2019; Trzepizur et al. 2022; Martinez‐Garcia et al. 2023), but findings have been inconsistent. For example, Azarbarzin et al. (Azarbarzin et al. 2019) reported that HB is a more accurate indicator of SDB severity with respect to CCVE risk. In contrast, Sutherland et al. (Sutherland et al. 2022) found no added predictive value of HB over traditional metrics, and a study of subclinical myocardial injury noted that novel desaturation indices (including HB) offered no significant advantage over the AHI (Sigurdardottir et al. 2022).

Notably, several randomized trials (RICCADSA (Peker et al. 2016), SAVE (McEvoy et al. 2016), ISAACC (Sánchez‐de‐la‐Torre et al. 2020)) did not show cardiovascular benefit of CPAP therapy in patients selected by AHI alone. However, a post hoc analysis by Pinilla et al. (Pinilla et al. 2023) of the ISAACC trial demonstrated that integrating AHI and HB can identify patients who do derive benefit from CPAP. Specifically, patients with both high AHI (≥ 15/h) and high HB (≥ 73.1% min h^−1^) had significantly reduced cardiovascular events with CPAP, whereas those with high AHI but low HB did not. Similarly, data from the Sleep Heart Health Study and MrOS cohorts (Azarbarzin et al. 2020) (*n* > 7000) showed that only the combined elevation of AHI and HB independently predicted incident heart failure, outperforming either metric alone. These findings align with our results and extend the value of the AHI+/HB+ phenotype to the domain of AFDAS. By integrating two complementary SDB metrics, our study highlights the potential of this combined approach to guide targeted AF surveillance in stroke survivors with SDB.

One major strength of our study is the systematic implementation of prolonged cardiac rhythm monitoring (at least one 7‐day Holter ECG) as a standard diagnostic procedure at our centre. This protocol provides a higher diagnostic yield for AF compared to conventional in‐hospital monitoring. Indeed, prolonged monitoring has been shown to detect AF in a significant proportion of stroke/TIA patients that would be missed by shorter monitoring durations (Tsivgoulis et al. 2022). Our findings underscore the benefit of systematically applying extended ECG monitoring in stroke patients, particularly those with SDB.

Current guidelines support prolonged arrhythmia monitoring after stroke. The ESC guidelines (Hindricks et al. 2021) recommend using extended noninvasive or implantable monitors for AF detection in cryptogenic stroke patients, and the AHA/ASA stroke prevention guidelines suggest long‐term ECG monitoring when no contraindication to anticoagulation exists (Kleindorfer et al. 2021). However, the optimal duration and modality of monitoring remain unclear, indicating the need for further standardization. In addition, the latest European stroke‐related societies' joint statement emphasizes screening for SDB in stroke patients and using SDB severity to identify those at high risk for new CCVE (Bassetti et al. 2020). Our results support this approach, suggesting that combined AHI and HB assessment can refine risk stratification beyond AHI alone.

Accordingly, we benchmarked HB against established oximetry‐derived descriptors. In covariate‐adjusted models, classical saturation‐summary measures (mean SpO_2_, lowest SpO_2_, and T90) were not independently associated with AFDAS, whereas both ODI and HB were independently associated with AFDAS when modelled separately. However, discrimination was virtually identical (AUC ≈ 0.68), consistent with substantial physiological overlap between ODI (frequency‐based) and HB (dose‐based) hypoxemia measures in this cohort.

Finally, we explored whether HB's contribution varies across the spectrum of intermittent hypoxia severity. Although ODI and HB were strongly correlated overall, the correlation weakened at lower ODI values, indicating substantial interindividual variability in cumulative hypoxemic exposure among patients with similar desaturation frequencies. In ODI‐stratified analyses, HB remained independently associated with AFDAS when restricting the analysis to individuals with ODI ≥ 5 events h^−1^, whereas in the moderate‐to‐severe range (ODI ≥ 15 events h^−1^), HB neither improved discrimination nor provided incremental information beyond ODI. This pattern may suggest that, when desaturation frequency is modest, a dose‐oriented HB may better capture clinically relevant hypoxemic exposure.

Notably, the signal appeared to be driven primarily by individuals within the mild‐to‐intermediate range of intermittent hypoxia. Due to the limited number of events in narrower strata, further subdivision (e.g., isolating ODI 0–10 events h^−1^) was not feasible without compromising model stability. Therefore, these findings should be interpreted as hypothesis‐generating and warrant confirmation in larger cohorts with sufficient power for refined stratified analyses.

Several limitations should be acknowledged. First, RP in the acute stroke setting requires patient cooperation; individuals with reduced consciousness, medical instability, or oxygen supplementation were therefore less likely to complete RP, potentially introducing selection bias. Consequently, included patients were younger and had milder strokes (Table S1), indicating preferential inclusion of mild‐to‐moderate, clinically stable presentations. These feasibility constraints should be considered when interpreting the generalizability of our findings to more severely affected or clinically unstable post‐stroke populations.

Second, compared with full polysomnography, RP may underestimate SDB severity by relying on recording time rather than verified sleep time and by missing arousal‐related hypopnoeas. In addition, SDB severity may evolve after stroke. A meta‐analysis by Hasan et al. (2021) reported stable prevalence of mild SDB but declining rates of moderate and severe SDB from the acute to chronic phase, indicating that a single acute‐phase assessment may misclassify patients with transient SDB. Nevertheless, clinically relevant SDB persists in a substantial proportion of patients, supporting the relevance of early detection.

Third, the absence of electroencephalography precluded sleep‐stage assessment, and stroke‐related alterations in sleep architecture may further dilute event‐based indices, leading to conservative underestimation of HB. Given these methodological considerations and the absence of validated post‐stroke thresholds, HB was therefore dichotomized using the cohort median (35% min h^−1^) for internal risk stratification. Additionally, despite multivariable adjustment, residual or unmeasured confounding cannot be excluded. COPD, a possible source of non‐sleep apnoea‐related nocturnal hypoxemia and a comorbidity associated with AF, was rare in our cohort (*n* = 14 [1.5%]). This likely reflects the exclusion of patients receiving nocturnal supplemental oxygen, which precludes valid interpretation of hypoxemia‐derived metrics and preferentially underrepresents individuals with clinically relevant COPD. Consequently, confounding by COPD‐related hypoxemia is unlikely to account for the observed associations; however, generalizability to post‐stroke populations with higher COPD prevalence or routine oxygen therapy should be interpreted with caution.

A further limitation relates to the duration and modality of rhythm surveillance. Although our monitoring protocol, comprising up to three 7‐day Holter recordings within 6 months, represents an intensive post‐stroke monitoring strategy, it may still fail to detect very infrequent or late‐onset paroxysmal AF. Consequently, the incidence of AFDAS may have been underestimated. Continuous long‐term monitoring approaches, particularly implantable loop recorders, are likely to provide higher detection rates and may allow more precise risk stratification in this population. Finally, our observational design precludes causal inference that SDB directly promotes AF; interventional studies would be needed to confirm this link.

HB thresholds proposed in community‐based populations, such as HB > 60% min h^−1^ (Martinez‐Garcia et al. 2023), were derived under conditions of relatively stable sleep architecture and PSG‐based sleep‐time normalization, and their applicability to acute post‐stroke populations remains uncertain. In the present cohort, meaningful associations with AFDAS were observed at HB levels below these previously proposed thresholds. Accordingly, rather than applying an external absolute cut‐off, we used a cohort‐derived threshold (median HB; 35% min h^−1^) to enable internal risk stratification. This threshold is intended for within‐cohort comparison only and should not be interpreted as a definitive clinical decision threshold for post‐stroke patients.

Prospective multicentre studies are warranted to validate the combined AHI‐HB risk model and to assess its potential in guiding clinical management. A critical open question is whether stroke patients identified as high‐risk by our criteria would benefit measurably from intensified cardiac monitoring or earlier initiation of anticoagulation. Furthermore, randomized trials are needed to determine whether therapeutic interventions for SDB (e.g., CPAP) in patients with elevated AHI and HB can reduce the incidence of AFDAS or improve clinical outcomes, as suggested by subgroup analyses of previous trials (Teba and Yaggi 2013). As the evidence base expands, integrating SDB characteristics into stroke care pathways may enable a more precise and individualized approach to preventing AF‐related recurrent strokes.

## 5 Conclusion

The findings indicate that the combined use of AHI and HB enhances AFDAS risk prediction in patients with ischemic stroke or TIA who have SDB. Improved identification of high‐risk individuals may allow for earlier AF detection and the implementation of timely preventive strategies to reduce the risk of recurrent stroke. This integrated approach contributes to more effective secondary prevention, facilitating targeted screening and intervention, and thereby improving clinical outcomes. Further research, as well as within the framework of the Bern Sleep‐Stroke Registry, is warranted to validate these findings. The development of standardized predictive models that incorporate the multifaceted pathophysiological mechanisms of SDB will be essential to optimize AFDAS risk stratification and support personalized management strategies in clinical practice.

## Author Contributions

**Xiaoli Yang:** conceptualization, methodology, software, data curation, formal analysis, investigation, visualization, project administration, writing – review and editing, writing – original draft. **Julian Lippert:** conceptualization, investigation, data curation, visualization, funding acquisition, project administration, writing – original draft (sections), writing – review and editing. **Claudio L. A. Bassetti:** conceptualization, funding acquisition, supervision, project administration, writing – review and editing. **Irina Filchenko**, and **Sebastien Baillieul:** software, validation, writing – review and editing. **Elias Auer:** validation, writing – review and editing. **Christian M. Horvath, Corrado Bernasconi, Stefan A. Bauer‐Gambelli, Anne‐Kathrin Brill, David J. Seiffge, Tobias Reichlin, Urs Fischer, Marcel Arnold**, and **Markus H. Schmidt:** resources, methodology, project administration, supervision, writing – review and editing.

## Funding

This work was supported by the Swiss National Science Foundation (SNF‐320030_149752), the Swiss Heart Foundation (FF19030), the Bern Medtech Collaboration Call 2024 (BMCC_2405), and a PhD fellowship from the China Scholarship Council, 202108170016 (to Xiaoli Yang).

## Ethics Statement

This work was approved by the Ethics Committee of the University of Bern (Protocol number: 2021‐00883).

## Consent

Individual informed consent was waived according to the Swiss law.

## Conflicts of Interest

The authors declare no conflicts of interest.

## Supporting information


**Table S1:** Baseline characteristics in patients included versus excluded.
**Figure S1:** Distribution of apnoea–hypopnoea index and hypoxic burden.
**Table S2:**. AFDAS adjusted odds ratios for oxygen desaturation index (ODI), mean saturation, lowest saturation, and T90 (percent time spent below oxygen saturation of 90%).
**Figure S2:** Scatterplots of log_2_(HB) versus ODI, displayed for ODI 0–15 and 15–60 events h^−1^. Points are stratified by AFDAS status, and the fitted LOESS curve summarizes the conditional trend across ODI values. The figure highlights greater dispersion of HB at lower ODI values, indicating heterogeneity in cumulative hypoxemic exposure among individuals with similar desaturation frequencies.
**Table S3:**. AFDAS adjusted odds ratios for AHI+/HB+ and covariates.

## Data Availability

The data that support the findings of this study are available on request from the corresponding author. The data are not publicly available due to privacy or ethical restrictions.
