# Supplementary material for: Enhanced Risk Stratification of Atrial Fibrillation Detected After Ischemic Stroke and TIA: The Role of Apnoea–Hypopnoea Index and Hypoxic Burden—A Study From the Bern Sleep‐Stroke Registry
Source: J Sleep Res. 2026 Apr 26;35(4):e70338. doi: 10.1111/jsr.70338 (PMC13357886; doi:10.1111/jsr.70338)
Supplement: Supplementary file 1 — Table S1: Baseline characteristics in patients included versus excluded. Figure S1: Distribution of apnoea–hypopnoea index and hypoxic burden. Table S2:. AFDAS adjusted odds ratios for oxygen desaturation index (ODI), mean saturation, lowest saturation, and T90 (percent time spent below oxygen saturation of 90%). Figure S2: Scatterplots of log2(HB) versus ODI, displayed for ODI 0–15 and 15–60 events h−1. Points are stratified by AFDAS status, and the fitted LOESS curve summarizes the conditional trend across ODI values. The figure highlights greater dispersion of HB at lower ODI values, indicating heterogeneity in cumulative hypoxemic exposure among individuals with similar desaturation frequencies. Table S3:. AFDAS adjusted odds ratios for AHI+/HB+ and covariates. [file JSR-35-e70338-s001.docx]

**Online Supplement**

**Table S1.** Baseline characteristics in patients included versus excluded

|  | **Excluded**  **n=4625 \| Missing n** | | **Included**  **n=911 \| Missing n** | | **Total**  **(n=5536)** | **P value** |
| --- | --- | --- | --- | --- | --- | --- |
| Age (mean, SD) | 72.7 (13.6) | 1 | 66.1 (13.6) | 0 | 71.6 (13.8) | < 0.001 |
| Male sex, n (%) | 2642 (57.1%) | 0 | 559 (61.4%) | 0 | 3201 (57.8%) | 0.018 |
| BMI | 26.3 (4.8) | 721 | 26.2 (4.4) | 22 | 26.3 (4.7) | 0.385 |
| Smoking status | 744 (20.3%) | 968 | 283 (31.1%) | 2 | 1027 (22.5%) | < 0.001 |
| NIHSS  at admission | 6.6 (7.5) | 161 | 3.4 (4.7) | 1 | 6.0 (7.2) | < 0.001 |
| Cardioembolic stroke | 1168 (32.0%) | 978 | 175 (19.2%) | 0 | 1343 (29.5%) | < 0.001 |
| Hypertension | 2761 (73.7%) | 877 | 616 (67.6%) | 0 | 3377 (72.5%) | < 0.001 |
| Diabetes | 795 (21.2%) | 880 | 172 (18.9%) | 0 | 966 (20.7%) | 0.117 |
| Dyslipidemia | 2560 (68.4%) | 884 | 612 (67.2%) | 0 | 3176 (68.3%) | 0.467 |

Comparison of baseline characteristics between patients included in the analytic cohort and those excluded from the source stroke/TIA population. Data are reported as mean (SD) or *n* (%); the adjacent column indicates the number of missing observations per group. Percentages were calculated using available-case denominators. *P* values reflect between-group comparisons using the statistical tests specified in the Methods section. †BMI, body mass index; NIHSS, National Institutes of Health Stroke Scale. Percentages are calculated among patients with available data for each variable; missing values were excluded from percentage and P-value calculations.

**Figure S1.** Distribution of apnea-hypopnea index and hypoxic burden


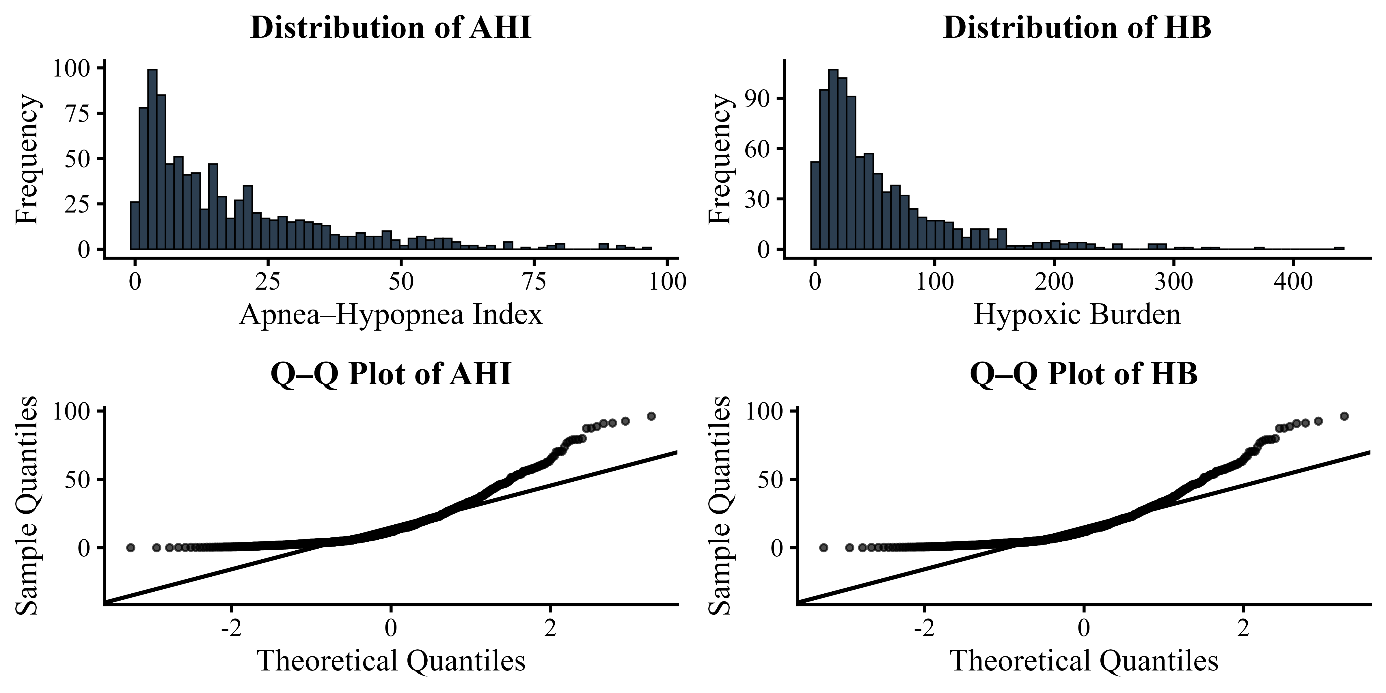


Empirical distributions of AHI and HB in the analytic cohort are shown as histograms (upper panels) and normal Q–Q plots (lower panels). The plots illustrate pronounced right-skewness and deviation from normality for both metrics.

**Table S2**. AFDAS adjusted odds ratios for oxygen desaturation index (ODI), mean saturation, lowest saturation, and T90 (percent time spent below oxygen saturation of 90%)

| Variables | Adjusted Odds Ratio (95% CI) | P values |
| --- | --- | --- |
| ODI | 1.02 (1.006, 1.03) | **0.003** |
| ODI (log-transformed, base=2) | 1.27 (1.08, 1.53) | **0.004** |
| ODI (per 5 events·h⁻¹) | 1.13 (0.71, 1.80) | 0.62 |
| HB | 1.004 (1.001, 1.008) | **0.008** |
| HB (log-transformed, base=2) | 1.22 (1.05, 1.43) | **0.01** |
| T90 | 1.004 (0.995, 1.01) | 0.33 |
| Mean saturation (%) | 0.99 (0.90, 1.10) | 0.89 |
| Lowest saturation (%) | 1.01 (0.99, 1.04) | 0.38 |
| ***Covariates:** age, sex, BMI, smoking, hypertension, diabetes, dyslipidemia, coronary heart disease, COPD, and heart failure | | |

Fully adjusted logistic regression models estimating odds ratios (ORs) for AFDAS associated with ODI and HB (untransformed and log₂-transformed) and for saturation-summary measures (T90, mean SpO₂, and lowest SpO₂). ORs are reported with 95% confidence intervals and corresponding *P* values; log₂-transformed estimates represent the change in odds per doubling of the exposure. Models were adjusted for age, sex, BMI, smoking, hypertension, diabetes, dyslipidemia, coronary heart disease, COPD, and heart failure.

**Figure S2.** Scatterplots of log₂(HB) versus ODI, displayed for ODI 0–15 events·h⁻¹ and 15–60 events·h⁻¹. Points are stratified by AFDAS status, and the fitted LOESS curve summarizes the conditional trend across ODI values. The figure highlights greater dispersion of HB at lower ODI values, indicating heterogeneity in cumulative hypoxemic exposure among individuals with similar desaturation frequencies.


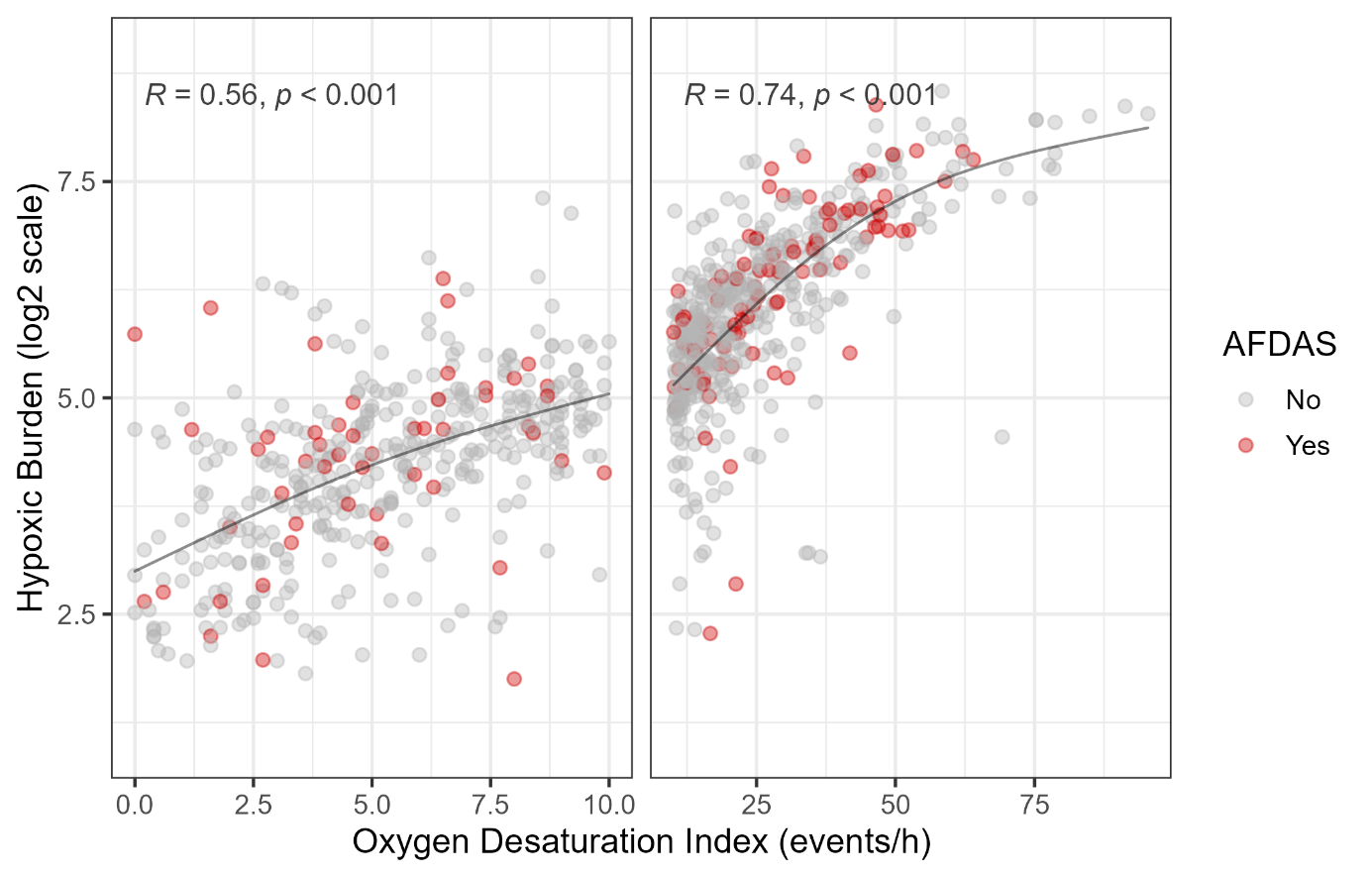


**Table S3**. AFDAS adjusted odds ratios for AHI+/HB+ and covariates

| Variables | N | AFDAS | Adjusted Odds Ratio (95% CI) |
| --- | --- | --- | --- |
| Age, per 5 years | 911 | 145 | **1.10 (1.01, 1.20)*** |
| Female sex | 352 | 62 | 1.26 (0.84, 1.90) |
| BMI | 911 | 145 | 0.99 (0.94, 1.03) |
| Smoking status | 283 | 30 | **0.51 (0.31, 0.81)**** |
| Hypertension | 616 | 108 | 1.05 (0.65, 1.72) |
| Diabetes | 172 | 27 | 0.74 (0.44, 1.21) |
| Dyslipidemia | 612 | 93 | 0.78 (0.52, 1.18) |
| Coronary Heart Disease | 124 | 30 | **1.75 (1.03, 2.93)*** |
| Chronic Obstructive Pulmonary Disease | 14 | 4 | 2.74 (0.71, 8.81) |
| Heart failure | 53 | 16 | 1.80 (0.90, 3.49) |
| AHI-/HB- | 404 | 46 | 1.00 |
| AHI+/HB+ | 336 | 73 | **1.97 (1.25, 3.12)**** |
| AHI+/HB- | 54 | 8 | 1.39 (0.57, 3.09) |
| AHI-/HB+ | 117 | 18 | 1.20 (0.63, 2.21) |

Fully adjusted logistic regression model reporting adjusted ORs (95% CI) for AFDAS for prespecified clinical covariates and for combined AHI/HB phenotype categories, using AHI−/HB− as the reference group (AHI dichotomized at 15 events·h⁻¹; HB dichotomized at the cohort median, 35 %min·h⁻¹). Asterisks denote statistical significance as indicated in the table.
